# State of the Art of Fuzzy Methods for Gene Regulatory Networks Inference

**DOI:** 10.1155/2015/148010

**Published:** 2015-03-23

**Authors:** Tuqyah Abdullah Al Qazlan, Aboubekeur Hamdi-Cherif, Chafia Kara-Mohamed

**Affiliations:** ^1^Information Technology Department, Computer College, Qassim University, Buraydah 51452, Saudi Arabia; ^2^Computer Science Department, Computer College, Qassim University, Buraydah 51452, Saudi Arabia

## Abstract

To address one of the most challenging issues at the cellular level, this paper surveys the fuzzy methods used in gene regulatory networks (GRNs) inference. GRNs represent causal relationships between genes that have a direct influence, trough protein production, on the life and the development of living organisms and provide a useful contribution to the understanding of the cellular functions as well as the mechanisms of diseases. Fuzzy systems are based on handling imprecise knowledge, such as biological information. They provide viable computational tools for inferring GRNs from gene expression data, thus contributing to the discovery of gene interactions responsible for specific diseases and/or *ad hoc* correcting therapies. Increasing computational power and high throughput technologies have provided powerful means to manage these challenging digital ecosystems at different levels from cell to society globally. The main aim of this paper is to report, present, and discuss the main contributions of this multidisciplinary field in a coherent and structured framework.

## 1. Introduction

Life in all living organisms is perfectly controlled by rigorous processes, thus sustaining stability in highly complex ecosystems from cellular level to society. The unprecedented amount of data generated by the Human Genome Project and the powerful sequencing and microarray technologies has to be carefully managed.* Ecosystem management combines the structuring and understanding of ecological information to facilitate the decision making in order to meet the society goals* [1]. Among the key elements in the cellular ecosystem we address, we emphasize gene regulatory networks (GRNs) as suitable means for representing causal relationships between genes. It is now widely accepted that GRNs have a direct influence on the survival and development of living organisms. Furthermore, GRNs offer a useful support for the understanding of the cellular functions and the mechanism of the diseases because they are able to reflect all interactions between genes with their products that determine the spatial and temporal expression patterns of a set of genes [2]. Gene interactions research studies have provided several useful applications such as new drugs discovery that act on regulatory pathways and the development of tracking methods in the dynamics of disease evolution within cells. GRNs inference is based on determining the genes that affect the expression of other genes and on adequately describing these effects.

Several methods have been developed for inferring GRNs and producing hypotheses about the presence or absence of interactions among genes, hypotheses that can later be tested by laboratory experiments [3]. In this context, various models have been proposed, such as Boolean networks and Bayesian networks for discrete models and ordinary differential equations (ODEs) and weight matrices for continuous models [4]. The ODEs representation was the most common model used while the piecewise linear differential equations (PLDEs) have been proposed for some approximations and simplifying nonlinear models. At a higher level, we attempt to show how machine learning can help in developing better bioinformatics methods and tools in a coherent manner by focusing on soft computing methods [5], with fuzzy methods being one of these.

Although the gene regulation has an inherent fuzzy nature, most of GRN studies were based on crisp algorithms [6]. The main motivation for using fuzzy methods lies in the fact that uncertainty and imprecision are inherent in biological data. This is due to the nature of biological experiments themselves. Therefore, the fuzzy approach, as a qualitative computational approach, can be considered as a suitable formalism to deal with the imprecision intrinsic to many biological problems, knowing that GRN inference is one of the most challenging ones [7].

Fuzzy logic, as a fundamental component of the fuzzy approach, is a combination of various mathematical principles for representing the knowledge depending on a gradual degree of membership instead of crisp membership available in Boolean logic. Fuzzy logic consists of several sets that can be used to map a certain input to an output, a process referred to as fuzzy inference. The two most well-known inference techniques are those due to Mamdani and Tagaki-Sugeno. These models depend on using simple language of IF-THEN rules in the description of the required system response as a function of several linguistic variables. Furthermore, fuzzy models are extremely robust and the input data can work with small or no tuning at all. Indeed, in the fuzzy models, there is no need for accurate inputs since it is naturally imprecise. Conversely, when the number of inputs and outputs increases, its complexity unfortunately increases too. The main benefits of fuzzy methods are based on the generality of function estimators, clarity, modularity, ability to be explained, easy handling of uncertainty, and parallel processing of rules. On the other hand, the main limitations that restrict the use of these systems are the high computational costs, comprehensibility, and optimization. These limitations are inherent in bioinformatics settings [8].

Despite the fact that fuzzy systems present powerful framework for GRN inference, the corresponding literature is diversified and scattered in numerous journals, conference proceeding, and Web sites. It is urgent that these contributions be compiled in a coherent and structured body of knowledge; hence this paper attempts to bridge this gap.

The paper is organized as follows.  Section 2 describes the GRN inference problem and the fuzzy approach to solve it.  Section 3 discusses mainstream fuzzy methods used in the GRN inference and Section 4 is devoted to hybrid methods.  Section 5 summarizes the different methods.  Section 6 discusses an example of GRN inference for* Saccharomyces cerevisiae*. The paper ends with a conclusion highlighting the main contribution and pointing toward eventual further improvements.

## 2. GRN and Fuzzy Approach

In this section, the basic GRN inference process is outlined. To do so, the emphasis is made on the utilization of the fuzzy inference approach.

### 2.1. Main GRN Inference Issue

GRNs inference is based on determining the genes that affect the expression of other genes and on describing these effects. At the abstract level, genes are usually represented by nodes and eventually interacting with each other through arcs, representing casual interactions between genes. The arrow (*i*, *j*) indicates that gene *i* regulates gene *j*. The process of inference consists in finding suitable GRNs that reproduce available data and extends this knowledge by predicting relations not explicitly given by actual data. For reasons of simplicity and without loss of generality, most studies concentrate on directed acyclic graphs (DAGs).

**Figure 1:**
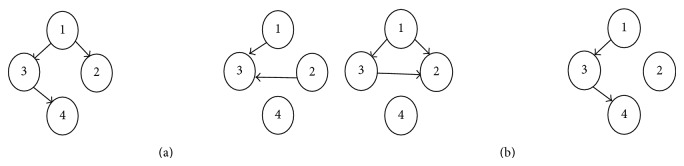
(a) Two GRN structures before inference. (b) GRN structures after evolvement during the inference process, whereby arcs are swapped between the nodes 3-4 and 2-3.

Figure 1(a) shows 2 hypothetical GRNs with 4 genes each.  Figure 1(b) shows the same 2 GRNs after evolvement during the process of inference. An increase (resp., decrease) of a cellular component, such as RNA (ribonucleic acid) or protein, in response to an external variable is called upregulation (resp., downregulation). No arc means that the genes are not directly correlated. We use −1 to indicate downregulation and 1 to show upregulation between genes in the inferred GRN, as shown in Figure 3.

### 2.2. Example of Inference

Figure 1 shows a 4-gene GRN to be improved through the inference process.

The two GRNs in Figure 1(a) represent two initial solutions whose links are generated by some process (e.g., randomly, by* a priori* knowledge, among others).

In Figure 1(b), the resulting GRNs are obtained after the inference process is achieved. A performance measure, such as a fitness function in evolutionary algorithms settings, is used to estimate the quality of the solution.

Additionally, Figure 3 shows another example of GRN, resulting from an inference process with cases of up- and downregulation for the yeast cell cycle.

### 2.3. Fuzzy Approach

#### 2.3.1. Steps of Fuzzy Approach

**Figure 2:**
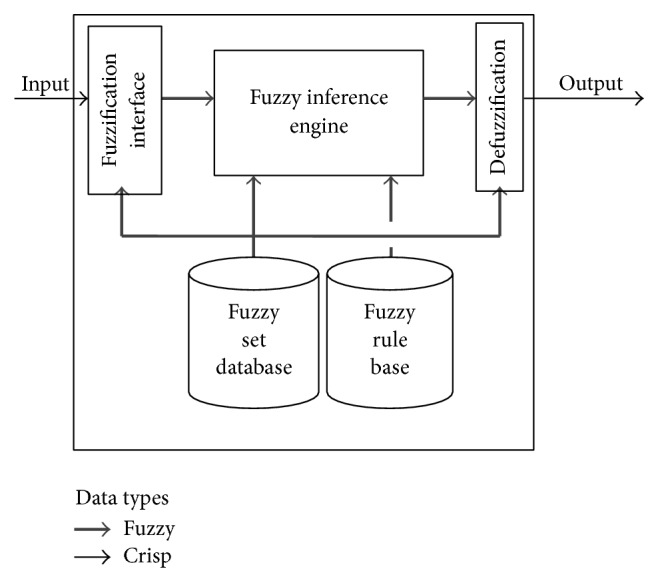
The basic fuzzy approach.

The basic fuzzy approach is depicted in Figure 2. Crisp values are given as inputs and crisp values are also obtained in the output after the fuzzy inference is accomplished. Fuzzification transforms crisp inputs and makes them amenable to inference by using fuzzy logic operators such as AND, OR, and NOT. Rules are evaluated and aggregated and then defuzzified to give a crisp result. As a summary, any fuzzy logic algorithm consists of three main traditional stages, fuzzification, inference, and defuzzification.

#### 2.3.2. Fuzzy Approach in GRN Inference

The general steps of fuzzy logic algorithm for inferring GRNs from microarray gene expression data are slowly emerging [4]. The main steps are described in Algorithm 1.

The fuzzy approach has an essential role in combining biology-based models with logical techniques for reconstructing the underlying GRN. Biological relations in the best-fitting fuzzy GRN models can productively recover both the direct and indirect interactions from preceding knowledge to offer best understanding of biological background about the transcriptional and regulatory mechanisms [9]. That is why one of the main methods that have emerged to infer GRNs is the fuzzy approach model, which can provide a basic rule structure due to the categorization of observations. These models are flexible and can be adjusted for several regulatory models and inferential rule groups. Furthermore, the nonlinear impacts between regulatory genes can be added, based on additional knowledge elicited from human specialists or automatically derived from specialized knowledge bases and converting data about gene relations into suitable fuzzy logic model.

Because the use of fuzzy logic allows the fusion of high-level, human-like reasoning in constructing and structuring the rule-based construction of the GRN, it is therefore advantageous for the biologist or subject matter expert who have extensive knowledge about existing regulatory mechanisms to express this information in an intuitive fashion using the semantics of fuzzy logic [4].

### 2.4. Complexity of Fuzzy Approach in GRN Inference

One of the main drawbacks of the fuzzy approach is its computational complexity. The Mamdani fuzzy inference involves a large computational burden. On the other hand, the computational efficiency of fuzzy inference can be improved using the Tagaki-Sugeno method, which has great attractiveness in control, especially for dynamic nonlinear systems, and works well with adaptive and optimization techniques [8]. One of the most challenging issues remaining is the intervention* via* an external control action on the GRN structure in order to enforce corrective therapeutic actions [10]. Unfortunately, with the number *n* of genes used for the model, the complexity of the inferred GRN increases in *O*(*n* ^3^), if we use a triplet of genes. More complex models such as a model with coactivators and corepressors would have an *O*(*n* ^5^) complexity. Accordingly, the algorithm can only focus on simple regulation patterns but cannot scale well to more complex models whose implementation time would be on the scale of years instead of hours.

Therefore, the computation time has to be improved. One way of doing so is by introducing a temporal gene expression clustering as a data processing step into the algorithm. In terms of computation time of experimental results for several real gene expression datasets, it is shown that the improved fuzzy approach is more efficient for reconstructing GRNs while the Mamdani model, although slow because of suffering from time complexity, has given the best performance in terms of the resilience to noise [7].

## 3. Discussion of Mainstream Fuzzy Methods in GRN Inference

In this section, we describe the mainstream methods in GRN inference based on the fuzzy approach, highlighting their merits and limits.

### 3.1. Detecting GRNs from Microarray Data

The appearance of microarray technology motivated computer scientists to improve algorithms for inferring GRNs and provided the possibility of simultaneously monitoring the expression levels for hundreds of thousands of genes. In addition, the DNA (deoxyribonucleic acid) microarray technology allowed the measurement of the RNA amount that is associated with a number of genes in parallel and decided which of them were expressed in a specific type of cells. This technology might face different types of difficulties in understanding the gene interaction. The basic reasons of these difficulties are the thousands of genes contained in microarray databases with few time steps in addition to the expression values that are subjected to noise [11].

Further, discovering GRNs using microarray data has some additional inherent difficulties. Various stages are used to regulate gene expression, including DNA transcription, RNA processing and transport, RNA translation, and posttranslational adjustment of proteins. The biological activity in the first of these stages is captured by DNA microarrays while the changes in mRNA transcript abundance do not necessarily reflect the regulation in the later stages. More experimental tests are therefore required for determining the validity of genetic interactions predicted by network models [4].

The use of fuzzy approach gave the ability to search for the potential regulatory interactions and comprehend reverse engineering of regulatory pathways related to a subset of interesting genes. The objectives of the proposed fuzzy approaches range from examining the entire genome of microarray data for small regulatory units to an exhaustive reconstruction of gene interactions in a certain pathway. These methods were effectively applied to both the simulated/experimental data related to the RAF (rapidly accelerated fibrosarcoma) signaling pathway and the real microarray data related to the yeast cell cycle, and the results were compared with other well-known algorithms. When making simulations, missing values were assigned using the* k*-nearest-neighbor (*k*NN) algorithm. Two ways were used to analyze the datasets.

First, eliminate all genes with expression levels below the noise threshold and that did not vary significantly during the cell cycle. Particularly, remove all genes with a maximum expression level below the first quartile in any of the four datasets or those that exhibited less than a threefold difference between maximum and minimum expression values in all four datasets. Using the fuzzy model, span the resulting dataset of 1737 genes and assess the gene triplets for enrichment of transcription factors and cell cycle regulated genes.

In the second analysis, select 12 genes from the yeast cell cycle pathway taken from KEGG (Kyoto Encyclopedia of Genes and Genomes http://www.genome.jp/kegg/) and run the fuzzy logic genetic search algorithm model on the gene expression values corresponding to these 12 genes. Check for consistency of the estimated GRN with the pathway depicted in KEGG. While the method is primarily used as a probing tool, the increase in computational overhead must be evaluated against the added flexibility of the model [4].

### 3.2. Predicting Changes in Expression Level

Fuzzy rules are used for finding gene regulation patterns from gene expression data using an activator-repressor GRN model. First, fuzzify gene expression profiles and then convert them into qualitative descriptors such as low, medium, and high. Then, a set of fuzzy rules is used to test all the probable combinations of triplets, target, repressors, and activators. The output obtained from the proposed system is then compared with the optimal gene expression level to discover whether they effectively fit the proposed fuzzy model. The difference between the achieved expression level and the target one is then used to rank the regulation combinations that have a small error and fit the majority of fuzzy rules. These combinations are inferred to exhibit the relations of the target, repressor, and activator combinations. An example of fuzzy rules is given in Algorithm 2.

Since the genes have three different states, the proposed method supports the concept of generalization in GRN inference by allowing a broader search space for inferring regulatory relationships and predicting changes in expression level of the target gene through interval time points. As a result, the expected regulation patterns of this model compose the GRN. The model is tested using yeast expression data that are obtained from* Saccharomyces cerevisiae* (yeast) cell cycle expression dataset [12].

#### 3.2.1. Fuzzy Clustering Method

Fuzzy logic can also be efficiently used for modeling the interaction and regulation of genes to precisely reflect the fundamental biology and to provide the complete analysis of GRN from clustering to evaluating the credibility of the network. A multiscale fuzzy clustering method using* k*-means algorithm allows the genes interacting among regulatory pathways across several conditions at various levels of detail. The causal relationships among groups of coregulated genes can be detected using fuzzy cluster centers. In this context, fuzzy method can measure the weight of expert knowledge and assist in quantifying uncertainty about the genes functions with the use of Gene Ontology (GO) (http://www.geneontology.org/) database in order to emphasize certain interactions.

The experiment on carbohydrate metabolism in the model plant (*Arabidopsis thaliana*) is used for gene expression to illustrate the proposed model. The information from the Gene Ontology (GO) database is used to evaluate the main gene regulatory relationships. A novel regulatory relationship about carbohydrate metabolism* trehalose* regulation was detected in the resulting network. The GRN inference algorithm has been efficiently evaluated to provide the information for time delay using the cluster centers. The feedback in the networks can also be allowed by using the algorithm that makes the results more reliable. The suggested work can be enhanced by incorporating the GRN model with accessible metabolic networks in the simulation of certain cellular processes [13].

#### 3.2.2. Fuzzy Approach in Modeling Gene Expression and Analyzing Protein Network

Because some genes interact between many regulatory pathways, soft clustering algorithm can be used for the description of these interactions. From all the reviewed applications in the study, it was found that the fuzzy approach-based models and fuzzy clustering techniques are powerful and efficient in many aspects and they are expected to play an essential role in integrating, analyzing, and modeling large amount of microarray datasets and complex biological systems.

Its prohibitive computational time remains one of the challenges because all the probable combinations of triplets must be examined. For example, the analysis of the relationships between 1,898 genes required 200 hours of computation on an 8-processor SGI Origin 2000 system. The results of experiments on many real gene expression datasets have illustrated the superior effectiveness for the improved fuzzy logic model in reconstructing GRNs in terms of computation time and the superior performance for Mamdani's model in terms of the ability for adapting to noise [7].

### 3.3. Inferring GRNs from Time Series Expression Data

Many developed analysis methods on reverse engineering of GRNs were proposed as a result of the increase in the number of time series gene expression experiments. The published methods evaluated all combinations of gene interactions in a gene expression time series database for those genes that directly fit a gene regulation. In order to reduce the computational cost, the search space of the least probable candidates is pruned in order to fit the required regulatory model. The method is used in evaluating numerous gene interactions for validating specific regulatory events based on filtering out the potential regulatory event candidates using fuzzy logic.

We can describe the changes that occurred in various expression levels and represent them as directional slope data while discarding combinations whose slope reaction is outside the proposed regulatory relation model scope. Several cheap hit vector calculations can be carried out to evaluate the ability of each gene relation triplet to fit in a more practical regulatory method.

The pruning process is performed effectively with the smooth response surface (SRS) algorithm on three* Saccharomyces cerevisiae* datasets, without significantly affecting the sensitivity of the SRS algorithm. The results gave 70% reduction in the computational time that was required for the processing of three datasets for* Saccharomyces cerevisiae* microarray time series [14]. However, it is not clear whether the proposed method can successfully be applied to more complex GRNs.

#### 3.3.1. Fuzzy Relational System for GRN Inference

Fuzzy techniques can be used to discover the GRNs from time series gene expression data. Fuzzy rules are designed depending on fuzzy set theory and expressing levels of gene. The fuzzy dependency relationships in noisy and high-dimensional data are also discovered. An artificial bee colony-based (ABC) search algorithm is presented to discover the potential constructions for a GRN which correctly fit the time series data and to discover the probable gene relations. Thus, the technique defines the fuzzy dependency measure between genes based on converting the values of the quantitative expression into linguistic terms, and the gene interaction is explained using a group of fuzzy relational matrices. The proposed technique is based on the fact that the measured time points are restricted. It uses an* Escherichia coli *SOS (when DNA is damaged, the SOS response is activated by stopping the cell cycle and inducing DNA repair and mutagenesis) DNA that includes around 30 genes regulated at the level of transcription. Results demonstrate that we are able to detect the genes that have an impact on a target gene, recognizing if the target gene is activated or inhibited, based on the detected dependency relations. Moreover, the simulation results for both real and artificial data show that this technique can effectively capture the GRNs' nonlinear dynamics and reveal the possible gene interaction relationship [15].

#### 3.3.2. Fuzzy Data Mining Technique for Inferring GRNs from Time Series Expression Data

Fuzzy data mining techniques are capable of extracting the high-dimensional and noisy expression data in order to construct relevant fuzzy sequential relations among genes. In addition, they are also capable of discovering the dependency of genes with each other and the possibility of a gene to be activated or inhibited. As an example, one technique can also predict the influence of genes on a target gene in an unseen sample. The proposed method is evaluated with the use of real expression data where the results demonstrate that the use of a fuzzy model for analyzing gene expression data is very efficient [3].

#### 3.3.3. Inferring GRNs from Expression Data by Discovering Fuzzy Dependency Relationships

The detected fuzzy dependency relations of genes can uncover the biologically significant gene regulatory relations, which can then be utilized in inferring the fundamental structures of these networks [16]. The proposed method can be enhanced by relying on the naturally parallel nature of this method in detecting the dependency relation. This improvement will in its turn make this method able to tackle highly complex gene datasets.

## 4. Discussion of Hybrid Methods for GRN Inference

The benefits of combining fuzzy logic with other methods (e.g., soft computing methods) are well recognized. Ultimately, fuzzy models can be optimized using hybrid techniques based on artificial neural networks and/or genetic algorithms. This improvement can be obtained at different levels, not only for forming membership functions but also for choosing rules to be used in the fuzzy logic system. We briefly describe examples of hybridized fuzzy methods.

### 4.1. Fuzzy Inference Systems (FISs) and ODEs

#### 4.1.1. ANFIS and ODEs

A model for representing a small GRN can be combined with the features of both fuzzy inference systems (FISs) and ordinary differential equations (ODEs) models. A neural network is used to train the FIS as a part of an adaptive network-based fuzzy inference system (ANFIS). The neural network is also used for allowing the output and membership functions from FIS to be adapted, based on the training data which in turn reduce the previous knowledge needed for modeling the phenomenon. The proposed model is used for describing the* lac operon *(*lactose* operon) in* Escherichia coli* and then compared with other similar models. (The operation of* lac operon* can be summarized as follows. When the glucose which is the main carbon source in the* Escherichia coli* is not present in the environment cell, it is then formed by the bacterium using the lactose. This process indicates that there is a regulatory operation that allowed synthesizing the required enzymes for obtaining glucose.) The fuzzy logic allowed the expression of the rules by linguistic labels for integrating the expert knowledge in an easy way. Thus, the fuzzy rules were used for describing the behavior of the* allolactose* based on two inputs: internal lactose and *β-galactosidase*. Therefore, three fuzzy sets were identified as low, medium, and high. Eight possible rules related to the two inputs are produced and implemented [17].  Figure 4 shows a typical profile of fuzzy expression levels used in fuzzy rules.

#### 4.1.2. Advantages of Integrating ANFIS and ODEs

The behavior of nonlinear system is similar to the one obtained with ODE and enhanced by the flexibility of fuzzy logic and the ability of training provided by ANFIS. The ANFIS network allowed the obtainment of all data needed for describing the transient state of implemented experiments and gave a large quantity of ODEs stable points. This indicates the flexibility of FIS and the significant training capability of the network. However, the training process of the ANFIS network caused errors. These latter ones were then compared with the outcome errors of the models based on ODEs. Further, the proposed PLDE-based model can be improved using optimization approaches and heuristic methods.

This work showed that the fuzzy approach proved to be an important tool due to its ability to characterize nonlinear systems, its human-like language to define knowledge, and its facility to include and edit fuzzy rules. To obtain a more powerful method, fuzzy approach can be supplemented by ANFIS for obtaining knowledge from experimental data in addition to ODEs.

#### 4.1.3. Disadvantages of Using ANFIS and ODEs

There seems to be an issue in making a tradeoff between acceptable representations of biological phenomena without compromising the viability of its computational implementation. In addition to that, there is an urgent need in maintaining a simple and comprehensible language that allows systems analysis from a qualitative standpoint.

#### 4.1.4. Comparison of Pure FIS versus ANFIS/ODEs

Table 1 summarizes the characteristics that are supported by FIS, on the one hand, and/or by ANFIS and ODEs, on the other hand.

### 4.2. Modeling GRNs Using Fuzzy Petri Network (FPN)

#### 4.2.1. Basic Fuzzy Petri Network (FPN)

Since GRN is a model of gene interactions at the expression level, the microarray technology has an essential role in the reconstruction of GRN. The GRN inference has challenged computer scientists to develop more enhanced algorithms for modeling the underlying regulatory relations among genes. One of these methods is the fuzzy Petri net (FPN) which consists of a fuzzy reasoning process that is able to search the microarray databases for activator-repressor regulatory relations between genes.

*(1) FPN Method*. The aim of the fuzzy Petri net (FPN) method is to construct a rule-based reasoning system that takes into account the gene triplets from the microarray data and searches for activator-repressor regulatory relationships among those genes. The proposed method uses the ways of predicting changes in expression level in the target gene based on input expression level. This method removed the probable false predictions from the traditional fuzzy model and offered an open search space to infer the regulatory relations. An activator-repressor relationship was shown by the genes that most likely fit the model. The main properties of the proposed method were its ability to explain the classical fuzzy reasoning procedure and the visualization of the structure of a rule-based fuzzy system. Furthermore, the method provides the reasoning stages required to predict changes in the gene expression levels for validation. The results show that the proposed method is suitable and practicable for predicting changes in expression level of the target gene. The proposed work can be enhanced by integrating neural network for modeling GRN [18].

*(2) Limits of FPN Method*. The main limitation of the FPN method is that the truth degree of a proposition and the confidence degree of a rule should both be determined in advance. The characterization of these two degrees usually relies on experts' experience. This latter might be unavailable or difficult to obtain. Even if the experience is available, the modeling will induce some uncertainty in the reasoning.

#### 4.2.2. Enhancing FPN through FRBPN

An extension of the FPN for modeling and analysis of a GRN uses a fuzzy reasoning Boolean Petri network (FRBPN) method and the dynamical behavior of gene. The proposed model was tested on a GRN responsible for the carbon starvation nutritional stress response in the bacterium* Escherichia coli* cells with the use of a comprehensive database. The results showed that there are six genes specified, with their interactions that play an important role in this process. Six modules are identified that correspond to the truth tables describing the Boolean behavior of each one of the regulatory entities in the nutritional stress response network of carbon starvation [19].

#### 4.2.3. Comparison of FIS versus FPN and FRBPN

See Table 2.

### 4.3. Other Hybrid Methods 

#### 4.3.1. AFEGRN

The adaptive fuzzy evolutionary GRN (AFEGRN) method is destined to infer GRNs using fuzzy approach. Specifically, it uses data distribution for automatically determining model parameters, for example, number of clusters for fuzzy *c*-means and estimation of Gaussian distribution algorithm. The method is tested for both normal and cancerous breast GRNs. The results are consistent with the biological knowledge and demonstrate that the differentially expressed (DE) genes were the cause of most of the cancers related to GRN changes. This result might corroborate the use of the AFEGRN framework for modeling any GRN [6].

#### 4.3.2. Coalesce GRN (CGRN) Reconstruction Framework

The need for cross platform GRN fusion is highly motivated by the noisy nature of microarray data and platform bias. Since the produced gene expressions by various platforms are not directly comparable, the cross platform GRN fusion becomes a difficult task. The coalesce GRN (CGRN) reconstruction framework integrates a cross platform GRN in order to eliminate the experimental and platform bias with the use of Dempster-Shafer theory of evidence. The common cancer related regulatory links in 10 databases are found using the CGRN framework. These databases resulted from several microarray platforms, like Affymetrix (http://www.affymetrix.com/estore/) and cDNA arrays. The results illustrate that the proposed CGRN framework can efficiently be applied for cross platform GRNs fusion to find out tumor specific links and unknown pathways [21].

#### 4.3.3. Combining Fuzzy Clustering and Bayesian Networks for Modeling GRNs

The Bayesian network (BN) approach is mainly used in the expectation of the GRNs from the expression data. Two main problems deeply undermine the efficiency of BN methods. One is the excessive computational time. The other major issue is the search space of possible BNs, which is very large because of the very large number of genes. Due to the fact that both the hierarchy and modularity are main biological networks features, global networks can be gained by collecting local components which consist of genes that have the same function and may have the same or a similar expression pattern. Combining fuzzy clustering and Bayesian networks for modeling is used in order to reduce the search space. In the learning process of local networks, the existence of gaps among the number of genes and samples is minimized and the search space is reduced. Simulation results confirm that the proposed approach can effectively predict GRNs and obtain noticeable improvement in the accuracy and the reduction of computational time in comparison with other available BN approaches [11].

#### 4.3.4. Inferring Fuzzy Cognitive Maps for GRNs

Fuzzy cognitive maps (FCMs) are used for representing GRNs. The method is based on an ant colony optimization (ACO) and relies on a decomposed method for reducing the dimension of the problem. Thus, the FCM learning algorithm becomes more scalable. Gene expression data are represented by fuzzy variables, and the relationships between genes are modeled through the fuzzy relations in FCMs.

Since the previously proposed FCM learning algorithms can only learn less than 40 nodes, the extended algorithm is able to learn FCMs with more than a hundred nodes. It can also avoid the discretizing of expression data, allowing the representation of genes dynamics more accurately. The basic problem of optimizing the whole weight matrix is reduced to smaller optimizing problems of one column of the weight matrix. Evaluation is done using 10 DREAM-4 datasets, from the DREAM Project (http://www.the-dream-project.org/). The results show that the FCM-based method outperforms other methods such as ODEs and Bayesian networks in part of the 10-gene network problems and in all of the 100-gene network problems. The suggested work can be further developed for application to larger networks [2].

#### 4.3.5. Comparison of FIS and Other Hybrid Methods

See Table 3.

## 5. Summary of Fuzzy Contributions for GRNs Inference

The main contributions of GRN inference based on the fuzzy approach and its extensions are summarized in Table 4.

## 6. Example of GRN Inference

**Figure 3:**
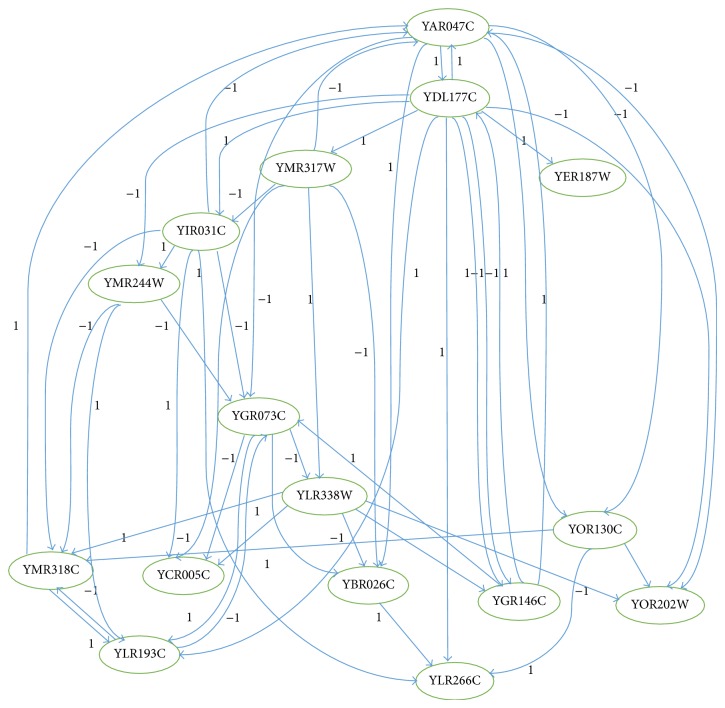
Inferred GRN of yeast cell cycle genes dataset with 16 genes and 50 direct links showing upregulation and downregulation. The notation (−1) is used to indicate downregulation and (1) to show upregulation between genes.

**Figure 4:**
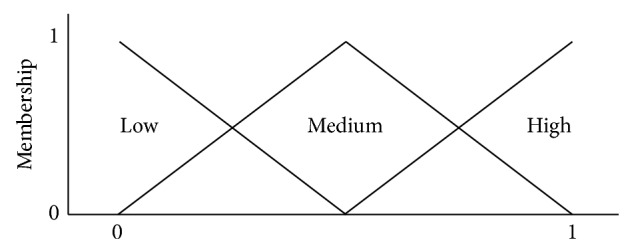
Example of fuzzy expression levels.

The biograph in Figure 3 shows genes interactions in the dataset of* Saccharomyces cerevisiae* (yeast) showing 16 genes represented as nodes and gene-gene interactions drawn as 50 links (directed edges) between genes. For example, gene YAR047C is

- downregulated by genes YIR0317C and YMR317W (−1 on the arrow);
- upregulated by genes YDL177C and YMR318C (1 on the arrow).

## 7. Conclusion

Fuzzy approach provides useful means for GRNs inference as reported in the diversified methods described in this paper. This is due to the fact that the underlying biological processes are best described using approximate features and not crisp values. The main contribution of the paper is that it gives an up-to-date hierarchical structuring of the various applications of fuzzy systems to GRNs; contributions now spread in diversified and scattered literature. We focus not only on the general concepts of fuzzy systems but also on their effective applications in GRNs inference, stressing both merits and limitations of each method. However, we do not enclose any biological aspects of the methods described or case studies on particular datasets as these are detailed in each method referenced. Despite the computational power offered by fuzzy systems in dealing with GRNs inference, better results are obtained using fuzzy method combined with other soft computing methods such as neural networks, genetic search, and Petri nets, among others. An interesting avenue for future research is to rank all the methods described above and others in terms of suitability for GRN inference.

## Figures and Tables

**Algorithm 1:**
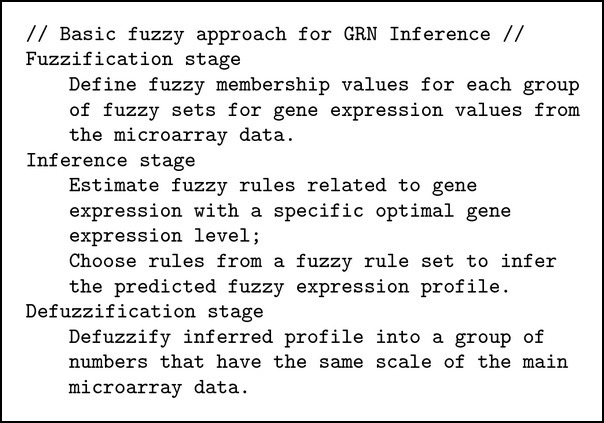
Fuzzy approach in GRN inference.

**Algorithm 2:**
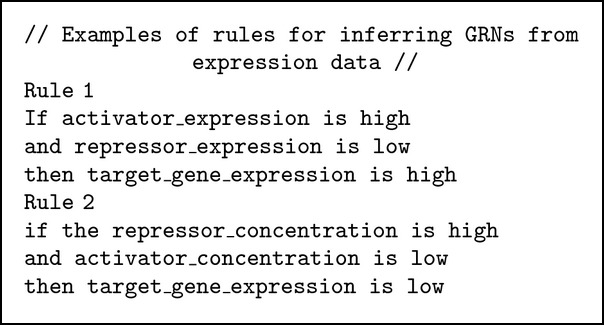
Predicting changes in gene expression level.

**Table 1:** Comparison of pure FIS versus ANFIS and ODEs.

Characteristics	Methods	
	FIS	ANFIS/ODEs
Characterization of nonlinear systems	Yes	Yes

Automatic training/learning	No	Yes

Reduction of knowledge needed for modeling biological phenomenon	Needs human expert. It is tedious and scarce	Automatically done directly from datasets

Adaptation of output and membership functions from FIS	No adaptation	Automatic adaptation

**Table 2:** Comparison of pure FIS versus FPN and FRBPN.

Characteristics	Methods		
	FIS	FPN	FRBPN
Predicting changes in expression level of the target gene	No	Yes	Yes

Need determination of truth degree of a proposition in advance	Yes (need of human expert)	Yes (need of human expert)	No (no need of human expert)

Need determination of the confidence degree of a rule in advance	Yes (need of human expert)	Yes (need of human expert)	No (no need of human expert)

**Table 3:** Comparison of FIS versus other hybrid methods.

Characteristics	Methods				
	FIS	AFEGRN	CGRN	Fuzzy clustering and BNs	FCMs
Automatic model parameters estimation, for example, number of clusters for fuzzy *c*-means	No	Yes	NT^*^	NT^*^	NT^*^
Cross platform GRNs fusion	No	NT^*^	Yes	NT^*^	NT^*^
Eliminate the experimental and platform bias	No	NT^*^	Yes	NT^*^	NT^*^
Reduction of search space/complexity	No	No	NT^*^	Yes (better than traditional BNs)	Yes (better than BNs and ODEs)

^*^NT means “not tested” (information not available): the corresponding method has not been tested against the specific characteristic mentioned in Table 3.

**Table 4:** Main fuzzy methods in GRN inference.

Dataset	Modeling method	Tool/technique software/database	References
*Saccharomyces cerevisiae* (yeast)	Fuzzy logic + clustering	Preprocessing algorithm coded in MATLAB	Ram et al. [9]

*Saccharomyces cerevisiae* (yeast) cell cycle	Fuzzy rules	GeneChip + SAGE	Woolf and Wang [12]

*Saccharomyces cerevisiae *	Fuzzy logic + clustering	DSOM approach + ART	Ressom et al. [20]

Breast cancer (cancerous cells in human)	AFEGRN	AFEGRN framework	Sehgal et al. [6]

10 different cancer datasets (cancerous cells in human)	Coalesce GRN (CGRN)	Cross platform GRN fusion	Sehgal et al. [21, 22]

Bacterium *Escherichia coli *	FRBPN	FRBPN technique	Hamed [19]

*Saccharomyces cerevisiae *	Combined fuzzy clustering and Bayesian networks (FCBN)	Software platform MATLAB and BNT package	Wang et al. [11]

*Lac operon* in *Escherichia coli *	Combined ODEs models and FIS	Simulink, fuzzy logic Toolbox and Optimization Toolbox in MATLAB	Muñoz et al. [17]

*Arabidopsis thaliana* plants, Affymetrix *Arabidopsis* ATH1 genome with 22K genes	Fuzzy cognitive map (FCM) model + clustering	FCModeler tool	Du et al. [13]

*Escherichia coli* bacteria SOS DNA consisting of 30 genes	Fuzzy logic + artificial bee colony (ABC)	ABC- and DE-based simulations, on Pentium	Das et al. [15]

*Saccharomyces cerevisiae *	Fuzzy Petri net (FPN)	FPN graphical tool	Hamed et al. [18]

*Saccharomyces cerevisiae *	Fuzzy logic based modeling	Fuzzy data mining technique	Ma and Chan [3]

*Escherichia coli* and *Saccharomyces cerevisiae* (210 time points in 100-gene networks), 10 DREAM-4 datasets	Fuzzy cognitive maps (FCMs) model and ant colony optimization (ACO)	Simulation using stochastic differential equations, DREAM project	Chen et al. [2]

*Saccharomyces cerevisiae* microarray time series (three datasets)	Activator-repressor regulatory model, SRS biological model	SRS program, regulatory-fit scoring method	Volkert and Malhis [14]

*Saccharomyces cerevisiae* genes time series gene expression data (about 800 cell cycle regulated genes)	Fuzzy data mining model	C4.5, SVM, and FID, 10-fold cross validation strategy	Ma and Chan [16]

Yeast cell cycle (cell-synchronized datasets with 14 time points)	Fuzzy logic genetic search algorithm model	KEGG database, *k*-nearest-neighbor (*k*NN) algorithm	Brock et al. [4]
